# 978. Expanding Burn Care Beyond the Acute Setting: Outreach Education for Rehabilitation and Home Health Nurses

**DOI:** 10.1093/jbcr/irag033.575

**Published:** 2026-04-06

**Authors:** Viviana Rubio, Vitina Kammin, Haaris Mir, Jonathan D Freedman, Sean Benoit

**Affiliations:** HCA Florida Kendall Hospital, Miami, FL; HCA Florida Kendall Hospital, Miami, FL; HCA Florida Kendall Hospital, Miami, FL; HCA Florida Kendall Hospital, Miami, FL; HCA Florida Kendall Hospital, Miami, FL

## Abstract

**Introduction:**

Burn survivors often transition from acute care to rehabilitation centers and home health agencies, where wound management remains critical to recovery. Nurses in these settings may have limited exposure to complex burn injuries, leading to uncertainty in practice and gaps in care. To address this, our ABA-verified burn center implemented an outreach education initiative to enhance nurse confidence, standardize wound care practices, and strengthen communication between community providers and burn specialists.

**Methods:**

Structured education sessions were developed and delivered to rehabilitation and home health nurses, with training now completed at nine post-acute care facilities where our burn patients are most frequently referred. The curriculum focused on wound assessment, dressing selection and application, pain management, infection prevention, and recognition of complications requiring escalation. Educational strategies included demonstrations, case-based discussions, and hands-on practice with burn-specific materials. To reinforce learning, direct communication pathways with the burn team were established to support ongoing collaboration after training.

**Results:**

Feedback from participants demonstrated improved comfort with complex dressing changes and greater consistency in wound care practices. Nurses reported increased confidence in escalating concerns, and facilities noted stronger collaboration with the burn center. The initiative improved both technical skill and interprofessional communication across the continuum of care.

**Conclusions:**

This outreach education initiative successfully expanded burn expertise into the post-acute care setting. By completing training at nine facilities, our program strengthened community capacity to provide specialized wound care, promoted continuity across transitions, and enhanced patient-centered recovery.

**Applicability of Research to Practice:**

This model of partnership has the potential to decrease rehospitalizations, improve long-term outcomes for burn survivors, and provide a replicable framework for collaboration between burn centers and community agencies nationwide.

**Funding for the study:**

N/A.

